# Evaluating the efficacy of moving cupping for chronic low back pain using shear wave elastography: A parallel-arm, randomized controlled trial

**DOI:** 10.1097/MD.0000000000034067

**Published:** 2023-08-04

**Authors:** Yeonhak Kim, Jihun Kim, Taewook Lee, Eunseok Kim, Kun Hyung Kim, Gi Young Yang

**Affiliations:** a Department of Acupuncture and Moxibustion Medicine, Pusan National University Korean Medicine Hospital, Mulgeum-eup, Yangsan-si, Gyeongsangnam-do, Korea; b Division of Clinical Medicine, School of Korean Medicine, Pusan National University, Mulgeum-eup, Yangsan-si, Gyeongsangnam-do, Korea.

**Keywords:** chronic low back pain, complementary therapy, Korean medicine, moving cupping, muscle relaxation, noninvasive treatment, pain management, randomized controlled trial, shear wave elastography, ultrasonography

## Abstract

Chronic low back pain (CLBP) is a common condition that affects millions of people worldwide. Moving cupping has gained popularity as a complementary therapy for managing CLBP owing to its noninvasive and cost-effective nature. However, the lack of objective measures to assess its therapeutic effect has been a considerable challenge in evaluating the effectiveness of moving cupping for CLBP management.

**Methods:**

We developed a randomized controlled trial (RCT) protocol for evaluating the effectiveness of a noninvasive treatment using moving cupping by assessing muscle relaxation with shear wave elastography (SWE). It involves the recruitment of 68 patients with CLBP and randomly assigns them to either the treatment or control group. The treatment group will receive moving cupping therapy for 2 weeks, while the control group will receive placebo treatment. It will utilize SWE to evaluate muscle relaxation at baseline, after 2 weeks of treatment, and 1 week after the end of treatment. Subjective reports of pain intensity and quality of life are also recorded at each time point.

**Discussion:**

The protocol developed here utilizes SWE to objectively measure muscle stiffness, and coupled with moving cupping therapy, may be effective in conveying relative comparisons before and after treatment. Moving cupping therapy is expected to promote muscle relaxation and pain relief in patients with CLBP. This study has the potential to contribute to the development of objective measures for evaluating the therapeutic effects of traditional therapies and to provide valuable insight into their efficacy.

## 1. Introduction

Chronic low back pain (CLBP) is a common condition that affects millions of people worldwide. It is defined as pain that persists for more than 3 months in the lower back region, and is one of the leading causes of disability and work absenteeism.^[1]^

Moving cupping is a traditional therapy that has been used for centuries to manage pain and promote relaxation. This therapy involves applying suction cups to the skin, which are subsequently moved around the body to promote blood flow and relieve muscle tension.^[2]^ Recently, moving cupping has gained popularity as a complementary therapy for managing CLBP owing to its noninvasive and cost-effective nature.^[3]^ It has been suggested that moving cupping may be particularly effective in treating CLBP by targeting muscle tension and improving circulation.^[4]^

One of the main challenges in evaluating the effectiveness of moving cupping for CLBP management is the lack of objective measures to assess its therapeutic effect. Although subjective reports of pain relief are commonly used to evaluate the efficacy of complementary therapies, they are often unreliable and may be influenced by placebo effects.^[5]^ Shear wave elastography (SWE) is a novel imaging technique that allows for the noninvasive assessment of muscle stiffness and relaxation. SWE provides a quantitative measure of muscle relaxation by measuring the speed of shear waves propagating through the muscle tissue, which can be used to evaluate the efficacy of moving cupping for CLBP management.^[6]^

The aim of this study was to develop a randomized controlled trial (RCT) protocol to evaluate the effectiveness of moving cupping for CLBP management using SWE to assess muscle relaxation. Specifically, it investigates whether moving cupping promotes muscle relaxation and alleviates pain in patients with CLBP. By providing objective measures of muscle relaxation and pain relief, this RCT protocol has the potential to significantly improve our understanding of the efficacy of moving cupping as a complementary therapy for CLBP. The findings may provide valuable insights for clinicians and researchers to optimize the clinical application of moving cupping and SWE for CLBP management, potentially improving patient outcomes and quality of life.

## 2. Methods

### 2.1. Ethics statements

Ethical approval for this study was obtained from the Pusan National University Medicine Hospital, National Clinical Research Center for Korean Medicine (PNUKHIRB-2022-12-002-001) on January 3, 2023. This clinical research protocol was based on the content registered and approved by the Clinical Research Information Service (http://cris.nih.go.kr) of the Republic of Korea (KCT0008282) on March 3, 2023.

### 2.2. Study design and population

This study aims to investigate the efficacy and safety of moving cupping therapy for chronic pain using a parallel-arm RCT. The target population will consist of patients with CLBP who have experienced pain for at least 12 weeks. The study will be conducted at the outpatient clinics of the Pusan National University Hospital and Korean Medicine Hospital.

To ensure blinding, the study will include only patients with CLBP who have no prior experience with moving cupping. The study will be conducted by independent practitioners, with one performing the moving cupping treatment and the other evaluating SWE independently. Each treatment will be administered 4 times (twice a week, for 2 weeks), and a follow-up evaluation will be conducted 1 week later.

This study was designed as a superiority trial with a level of significance set at α = 0.05, a type II error (β) of 0.2, and a power of the test of 80%. The sample size was calculated based on similar studies,^[7]^ with a 1:1 ratio between the intervention and control groups, and a dropout rate of 15% was considered in each group. Sixty-eight patients with CLBP will be randomly assigned to either the intervention or control group in sealed envelopes. After treatment, patients will undergo an evaluation.

#### 2.2.1. Participant recruitment.

Participants will be recruited according to a plan approved by the Institutional Review Board and the Clinical Research Information Service at Pusan National University Medicine Hospital, National Clinical Research Center for Korean Medicine. The study will be explained to the participants by their primary physicians and written informed consent will be obtained from those who choose to participate.

#### 2.2.2. Inclusion criteria.

1. Individuals aged 18 years or older but under 60 years of age.^[8]^
2. Individuals with symptoms of low back pain lasting for at least 12 weeks.^[9]^
3. Individuals with a body mass index (BMI) of <35 kg/m^2^.^[10]^
4. Individuals with a numeric rating scale (NRS) pain intensity score ≥4.^[11]^
5. Individuals who can understand the purpose and procedures of the study, and voluntarily decide to participate and provide written consent after receiving an explanation.

#### 2.2.3. Exclusion criteria.

1. Individuals with medical experience of moving cupping.
2. QIndividuals with medical experience of gold thread therapy.^[12]^
3. Individuals diagnosed with spinal tumors, spinal infections, or ankylosing spondylitis.
4. Individuals with a history of spinal surgery such as spinal fusion or laminectomy.
5. Individuals with factors that may affect hemostasis such as taking anticoagulants or antiplatelet agents, or having bleeding disorders.
6. Individuals with severe atopic dermatitis, keloid-prone skin, other skin hypersensitivities, or uncontrolled diabetic skin diseases.
7. Individuals with a substantial neuropsychiatric history or current neuropsychiatric disorders.
8. Individuals with a history of alcohol or substance abuse.
9. Pregnant or lactating women or women planning to become pregnant.

#### 2.2.4. Study protocol.

Table 1 presents the comprehensive study schedule. Each candidate will receive an explanation of the study protocol from a physician and is required to provide consent. Thereafter, they will be requested to sign an informed consent form before proceeding with the examination, as shown in Table 1. To allocate enrolled study participants to either the treatment or placebo group during visit 1, independent researchers will deliver sealed and opaque envelopes labeled consecutively with numbers, which have been randomly allocated by an independent statistician. The blinding will be maintained until submission of the paper. Before and after each treatment session, SWE will be conducted. Additionally, the physician will monitor the adverse events during each visit.

**Table 1:** Study schedule.

		Treatment phase				Follow-up phase
		1 wk		2 wk		3 wk
	Screening	Visit 1	Visit 2	Visit 3	Visit 4	Visit 5
Informed consent acquisition	√					
Confirmation of eligibility (inclusion and exclusion criteria)	√					
Vital signs measurement	√	√	√	√	√	√
BMI measurement	√					
Demographic survey	√					
Medical history survey for the back and other organs	√					
Clinical examination†	√					
Physical examination	√					
Random allocation		√				
Moving cupping		√	√	√	√	
Placebo		√	√	√	√	
Outlier detection		√	√	√	√	
Confirmation of concomitant therapy		√	√	√	√	√
Shear wave elastography (before)		√	√	√	√	
Shear wave elastography (after)		√	√	√	√	
Numeric rating scale (NRS)	√	√			√	√
Oswestry disability index (ODI)		√			√	√
EQ-5D index		√			√	√
Patient global assessment (PGA)					√	√
Adverse event monitoring		√	√	√	√	√
Dropout confirmation		√	√	√	√	√

BMI = body mass index.

### 2.3. Intervention

#### 2.3.1. Moving cupping.

During the intervention, the participants will assume a prone position on a plinth with both elbows flexed to 90° and their shoulders abducted to 120° with external rotation. Cushions will be placed under the pelvis and legs to increase comfort, reduce lumbar lordosis, and optimize transducer contact, thereby enhancing the accuracy of the measurements obtained during the intervention.^[13]^ Thereafter, moving cupping will be administered bilaterally at the L1-5 level spinous process, 4 to 8 cm from the midline, for 5 minutes on each side, with pressure between 18 and 20 kPa (135–150 mm Hg). After a 15-minute rest period, the stiffness of the superficial multifidus muscle will be measured in kPa using SWE.^[2]^

#### 2.3.2. Control group.

The same procedure will be performed as in section 2.3.1., except that it will be conducted at 0 kPa (0 mm Hg).

#### 2.3.3. Shear-wave elastography measurement.

After measurement by the evaluator using ultrasound equipment, all SWE images will be stored on a separate storage device by a different researcher. To analyze the images, a Q-box will be inserted into the SWE image to evaluate the mean shear modulus in kPa of all the muscle fibers within the region of interest. The Q-box spatially averages the propagation speed of each shear wave to determine the shear modulus of each area of the imaged tissue. The shear modulus values will be averaged for the selected tissue area by averaging all the shear modulus values. Once the appropriate Q-box position is determined, the shear modulus values will be recorded 12 times in each trial. To reduce bias, the maximum and minimum values of the 12 recorded shear modulus (kPa) values will be excluded, and the average of the remaining 10 values recorded.

#### 2.3.4. Adverse events.

Possible adverse effects related to moving cupping include local adverse reactions such as blister formation, bleeding, persistent bruising, ongoing pain, stiffness, burning sensation, and heat at the treatment site. All significant adverse events that occur between the time of signing the informed consent form and the end of the trial will be documented in the medical records and reported to the Clinical Research Review Committee.

#### 2.3.5. Relevant concomitant care and interventions.

If concomitant medication or treatment within the last 3 months that could potentially affect CLBP is identified during screening, a wash-out period of at least 2 weeks is required before proceeding. There are no relevant concomitant care or interventions permitted during the study.

### 2.4. Primary and secondary endpoints

The primary endpoint is 2 weeks after treatment compared to baseline (visit 1). The secondary endpoints are presented in Table 2.

**Table 2:** Secondary outcomes.

Assessment method	Objective	Endpoint
Shear wave elastography	To assess the efficacy of Moving cupping therapy	3 wk after baseline (1 wk post-treatment)
NRS	To assess the efficacy of Moving cupping therapy	3 wk after baseline (1 wk post-treatment)
ODI	To assess the efficacy of Moving cupping therapy	3 wk after baseline (1 wk post-treatment)
EQ-5D	To assess the efficacy of Moving cupping therapy	3 wk after baseline (1 wk post-treatment)
PGA	To assess the efficacy of Moving cupping therapy	3 wk after baseline (1 wk post-treatment)
Outlier detection	To assess the efficacy of Moving cupping therapy and reflect the psychological state of participating patients in the study	Visit 4 (twice a wk visit, 2 wk)
BMI	To evaluate the impact of obesity on Moving cupping therapy	Screening phase

BMI = body mass index, NRS = numeric rating scale, ODI = Oswestry disability index, PGA = patient global assessment.

### 2.5. Data collection and management

An electronically generated Case Report Form will be used and managed by qualified and authorized investigators and physicians. All the data recorded in the electronically generated Case Report Form will be consistent with those of the original material. During the study, the investigator will collect data at each visit. All study findings and records will be treated with strict confidentiality, and patients are to be identified solely by their patient number and/or birth date, and never by name. To protect participant anonymity, the investigator will maintain the confidentiality of the patient-identifying documents. The following data will be collected.

- Date of birth (age), sex, medical history, history of current disease, and pregnancy status.
- Body weight and height at first visit, including BMI calculations.
- Vital signs (body temperature [°C], blood pressure [mm Hg], pulse rate [beats/minute]) at every visit.
- SWE before and after each treatment.
- NRS score at screening and visits 1, 4, and 5.
- Oswestry Disability Index and EQ-5D index at visits 1, 4, and 5.
- Patient Global Assessment at screening visits, and visits 1, 4, and 5.
- Outlier detection for each procedure.

Additionally, an independent clinical research organization will conduct data management and data monitoring. Interim analyses will not be conducted, and the trial will be terminated by the principal investigator when the study process for all enrolled study participants has been completed.

### 2.6. Statistical analyses

The population to be analyzed is defined as all intervention and control groups that participate in the moving cupping study. Descriptive statistics will be used to summarize the demographic characteristics and baseline clinical data of the participants, including variables such as sex, age, BMI, medical history, and medication history by treatment group. All statistical analyses will be 2-tailed, with a significance level of 5%. Statistical software SAS Version 9.4 (SAS Institute, Inc., Cary, NC) will be used for the analysis. In the case of missing values, multiple imputations will be used for data processing.

## 3. Discussion

Previous studies on Korean medicine have predominantly relied on subjective measures for assessing treatment efficacy, limiting their ability to provide objective information on changes before and after treatment.^[14]^ To address this limitation, we used SWE in our protocol for evaluating the efficacy and safety of moving cupping combined with SWE for CLBP management. This approach allowed us to quantify changes in muscle stiffness as an objective measure of the therapeutic benefits of moving cupping. Our study is unique in that it evaluates the effectiveness of moving cupping in promoting muscle relaxation, pain relief, and healing through SWE, providing a novel approach to CLBP management. Additionally, the use of SWE in measuring muscle stiffness changes is a significant departure from previous Korean medicine studies, which primarily relied on subjective assessments.

In this study, we outlined a protocol for an RCT aimed at evaluating the efficacy and safety of moving cupping combined with SWE for CLBP management. The use of objective measures to assess complementary therapies is a significant challenge in the field, and this study aimed to address this issue by using SWE to assess muscle stiffness and relaxation as a quantitative measure of the therapeutic benefits of moving cupping.

As previously suggested, the degree of muscle contraction in the lower spine may vary depending on the underlying condition causing the low back pain, such as a herniated disc.^[15]^ Furthermore, CLBP and muscle stiffness are interrelated, with muscle stiffness being both a cause and consequence of CLBP.^[16]^

The use of this RCT protocol has the potential to significantly improve our understanding of the efficacy of moving cupping as a complementary therapy for managing CLBP and other musculoskeletal conditions. The findings may provide valuable insights for clinicians and researchers to optimize the clinical application of moving cupping and SWE in managing CLBP, potentially improving patient outcomes and quality of life.

This RCT protocol has several limitations. First, it is not double-blinded, which may introduce researcher bias. However, the protocol minimizes bias by taking the mean of 10 out of the 12 quantitative values recorded in ultrasound images and excluding the maximum/minimum values. Second, it is not an absolute comparison study, and variance may be high owing to individual differences in values. However, it evaluates the effectiveness of recovery through a relative value comparison before and after treatment, and explores the significance of value changes with qualitative evaluations such as the NRS. Third, it is a placebo-controlled study, and information bias may be introduced if the patient understands the difference in treatment. However, it controls for participants’ expectations by excluding those who have experienced moving cupping, omitting treatment information, or offering a sham treatment similar to the actual procedure.

There are several areas that warrant further research to determine the full scope and implications of this treatment. Long-term research on the efficacy of moving cupping and SWE in treating CLBP may provide additional insight into the potential for relieving and preventing symptoms. Additionally, exploring the therapeutic efficacy of this combined therapy for other conditions may open up new avenues for treating other diseases. Moreover, studying the combined therapy of moving cupping and SWE with other treatments may help identify the most effective approach to managing pain and improving function. Finally, providing clinical guidelines for the application of the RCT results may help healthcare professionals use this therapy more effectively to benefit their patients.

In conclusion, we present a RCT protocol for investigating the efficacy and safety of moving cupping combined with SWE for patients with CLBP. This therapy may have a significant effect on pain reduction and functional improvement. Such research avenues can expand our understanding of the potential benefits of moving cupping and SWE, and help optimize their clinical application.

## Author contributions

**Conceptualization:** Yeonhak Kim, Gi Young Yang.

**Data curation:** Yeonhak Kim, Jihun Kim, Taewook Lee.

**Formal analysis:** Yeonhak Kim, Eunseok Kim, Kun Hyung Kim.

**Funding acquisition:** Yeonhak Kim, Gi Young Yang.

**Investigation:** Yeonhak Kim, Jihun Kim, Taewook Lee.

**Methodology:** Yeonhak Kim, Eunseok Kim, Kun Hyung Kim.

**Project administration:** Yeonhak Kim, Gi Young Yang.

**Resources:** Yeonhak Kim.

**Software:** Yeonhak Kim.

**Supervision:** Gi Young Yang.

**Validation:** Eunseok Kim, Kun Hyung Kim, Gi Young Yang.

**Visualization:** Yeonhak Kim.

**Writing – original draft:** Yeonhak Kim.

**Writing – review & editing:** Yeonhak Kim, Jihun Kim, Taewook Lee, Eunseok Kim, Kun Hyung Kim, Gi Young Yang.
